# Epigallocatechin-3-Gallate Reduces Visceral Adiposity Partly through the Regulation of Beclin1-Dependent Autophagy in White Adipose Tissues

**DOI:** 10.3390/nu12103072

**Published:** 2020-10-08

**Authors:** Cheoljun Choi, Hyun-Doo Song, Yeonho Son, Yoon Keun Cho, Sang-Yeop Ahn, Young-Suk Jung, Young Cheol Yoon, Sung Won Kwon, Yun-Hee Lee

**Affiliations:** 1 College of Pharmacy and Research Institute of Pharmaceutical Sciences, Seoul National University, Seoul 08826, Korea; hgg121@snu.ac.kr (C.C.); hyundoo0186@gmail.com (H.-D.S.); syh8116@naver.com (Y.S.); ykc224@naver.com (Y.K.C.); 000043@naver.com (S.-Y.A.); yunyochl@snu.ac.kr (Y.C.Y.); swkwon@snu.ac.kr (S.W.K.); 2 College of Pharmacy, Pusan National University, Busan 46241, Korea; youngjung@pusan.ac.kr

**Keywords:** AMP-activated protein kinase signaling pathway, epigallocatechin-3-gallate, obesity, white adipose tissue

## Abstract

Epigallocatechin-3-gallate (EGCG) is a primary bioactive phytochemical in green tea. Its therapeutic potential in metabolic diseases has been reported; however, the molecular mechanisms of the anti-obesity effect of EGCG have not been fully elucidated. In this study, we examined the effects of EGCG on lipid metabolism and autophagy in adipose tissue. After 8 weeks of high-fat diet feeding, mice were treated with EGCG (20 mg/kg/day) for 2 weeks to test in vivo anti-obesity effects of EGCG. EGCG treatment improved glucose tolerance and caused body weight loss. Interestingly, reduced adipose tissue mass was more prominent in visceral compared to subcutaneous white adipose tissue. Mechanistically, EGCG treatment increased autophagic flux in white adipose tissue through the AMP-activated protein kinase-mediated signaling pathway. Adipocyte-specific knockout of Beclin1 mitigated the effects of EGCG on visceral adipose tissue mass and glucose tolerance, indicating that the anti-obesity effect of EGCG requires Beclin1-dependent autophagy. Collectively, our data demonstrated that EGCG has anti-obesity effects through the upregulation of Beclin1-dependent autophagy and lipid catabolism in white adipose tissue (WAT).

## 1. Introduction

Obesity, a worldwide health problem, is defined as abnormal fat accumulation and excess adiposity, and is generally caused by an imbalance between calorie intake and energy expenditure [1,2]. Notably, obesity is a risk factor that may cause chronic metabolic diseases, including type 2 diabetes and cardiovascular diseases [3].

Green tea is consumed throughout the world, and its various health benefits have been reported [4]. Research on green tea has shed light on the health-promoting effects of green tea catechin, including its ability to control hyperlipidemia and adiposity in rodent obesity model studies and clinical trials. The polyphenolic catechin, epigallocatechin-3-gallate (EGCG), is a principal bioactive constituent of green tea [4]. EGCG appears to have therapeutic potential against various diseases such as cancer, neurodegenerative and metabolic disorders [5]. With regard to effects on lipid metabolism, previous studies demonstrated that EGCG inhibits lipogenesis [6] and activates lipolysis [7] and non-shivering thermogenesis [8].

Adipose tissue accumulates excessive energy in adipocytes as triglycerides in lipid droplets (LDs) [1]. Negative energy balance, such as calorie restriction or fasting, activates lipolysis, LD breakdown, and produces free fatty acids (FFAs), which are provided through systematic circulation as an energy source [1]. Catecholamine is one of the primary promoters that regulate lipolysis through beta-adrenergic receptors in adipocytes [1]. Moreover, the cAMP-dependent protein kinase A-mediated downstream signaling pathway in this process promotes cytosolic lipase activation: hormone-sensitive lipase (HSL) and adipose triglyceride lipase (ATGL). Another important regulator of lipolysis and lipogenesis is the AMP-activated protein kinase (AMPK) signaling pathway [9]. By sensing the cellular deprivation energy state, AMPK activates catabolic processes that produce ATP, increase lipolysis [10] and fatty acid oxidation, but suppress ATP-expenditure anabolic pathways, lipogenesis [11,12]. In addition, AMPK induces autophagy by phosphorylating Unc-51 like autophagy activating kinase 1 (ULK1), an autophagy-initiating kinase [13]. In addition to cytosolic lipolysis, the autophagic breakdown of LDs, called lipophagy, involves lipid catabolism [14]. Although molecular mechanisms of LD-selective autophagy are not clear, this macroautophagic process contributes to LD breakdown in the liver and adipocytes [14]. We previously investigated the roles of EGCG in adipocytes and illustrated that EGCG treatment regulated autophagic lipolysis in adipocytes in vitro [15]. Thus, investigating the in vivo influence of EGCG on lipolysis and autophagy in adipocytes is significant to understand the anti-obesity mechanism by EGCG.

Here, we looked into the anti-obesity effects of EGCG using a high-fat diet (HFD)-induced obesity mouse model and demonstrated that EGCG treatment increased autophagy in visceral white adipose tissues (WATs). The induction of autophagy by EGCG was accompanied by the AMPK pathway activation. Further, the anti-obesity effects of EGCG treatment required Beclin1-dependent autophagy.

## 2. Materials and Methods

### 2.1. Animals

Male C57BL/6 mice were utilized in experiments following approved protocols by the Institutional Animal Care and Use Committees of Yonsei University (IACUC-201803-704-01) and Seoul National University (SNU-200406-1-1). Mice were raised on a 12-h light/12-h dark cycle and given free access to normal chow diet (NCD) and water at 22 ± 1 °C. Mice were acquired from Central Lab Animal Inc. (Seoul, South Korea). Adipoq-CreER (B6.129-Tg(Adipoq-cre/Esr1*)1Evdr/J: stock# 024671) mice were obtained from the Jackson Laboratory (Bar Harbor, ME, USA). Adipocyte-specific Beclin1 knockout (KO) mice were produced by crossing Adipoq-CreER mice and *Becn1*-floxed mice, as previously described [16]. Cre recombination was induced by tamoxifen (75 mg/kg/day, Cayman Chemical, Ann Arbor, MI, USA) administration dissolved in sunflower oil (Sigma, St. Louis, MO, USA) by oral gavage for 5 days.

High-fat diet (HFD) (Research Diets #D12492, fat: 60% kcal, energy protein: 20% kcal, carbohydrate: 20% kcal density: 5.21 kcal/g, New Brunswick, NJ, USA) were fed for 8 weeks. EGCG (20 mg/kg/day, Sigma, St. Louis, MO, USA) dissolved in distilled water were treated for 2 weeks by oral gavage [17,18]. The intraperitoneal glucose tolerance test was conducted through a method described previously [19]. EGCG was detected in the plasma samples obtained from the mice treated with EGCG or vehicle controls, as described in Supplemental Methods.

### 2.2. Quantitative PCR and Immunoblot Analysis

Quantitative PCR (qPCR) analysis was conducted through a method described previously [5]. Briefly, total RNA was extracted by utilizing TRIzol^®^ reagent (Invitrogen, Carlsbad, CA, USA). The High-capacity cDNA Reverse Transcription kit (Applied Biosystems, Foster City, CA, USA) was applied for cDNA synthesis, and qPCR was detected using iQ SYBR Green Supermix (Bio-Rad, Hercules, CA, USA). Primers for qPCR are described in Table S1.

Immunoblot analysis was conducted through a method described previously [20]. Proteins were detected with primary and HRP-conjugated secondary antibodies in Immunoblot analysis: α/β-tubulin (rabbit, CST, Danvers, MA, USA), AMPK (rabbit, CST, Danvers, MA, USA), phosphor-AMPK (rabbit, CST, Danvers, MA, USA), ATGL (rabbit, CST, Danvers, MA, USA), phosphor-ATGL (Ser406) (rabbit, Abcam, Cambridge, UK), LC3A/B (rabbit, CST, Danvers, MA, USA), FASN (rabbit, CST, Danvers, MA, USA), phosphor-SREBP1c (Ser372) (rabbit, CST, Danvers, MA, USA) and SREBP-1 (mouse, Santacruz, Dallas, TA, USA).

### 2.3. Histology

Hematoxylin/eosin (H&E) staining was conducted using paraffin sections, as described previously [16]. For the measurement of adipocyte diameters, H&E stained adipose tissue paraffin sections were used.

### 2.4. Statistical Analysis

For statistical analysis, we used Prism 5 software (GraphPad Software, La Jolla, CA, USA). Data were shown as mean ± SEM. An unpaired *t*-test was used between two groups to measure statistical significance.

## 3. Results

### 3.1. Two Weeks of EGCG Treatment Reduced Body Weight and Improved Glucose Tolerance in HFD-Fed Mice

**Figure 1:**
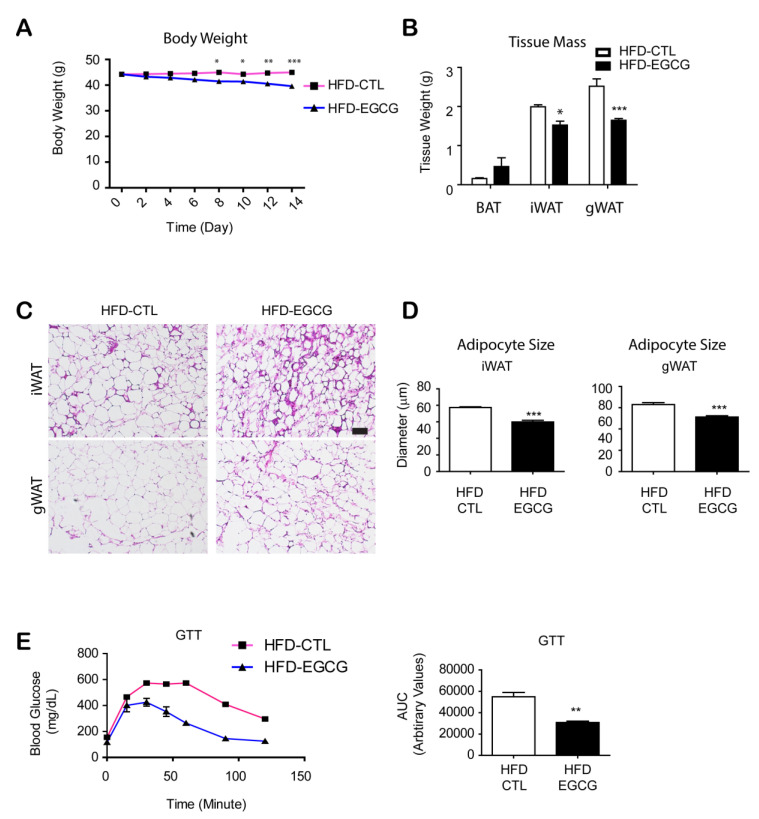
Effects of epigallocatechin-3-gallate (EGCG) on body weight and glucose tolerance of mice fed a high-fat diet (HFD). Mice were fed a high-fat diet (HFD) for 8 weeks and then treated with EGCG (20 mg/kg/day) (HFD-EGCG) or vehicle (HFD-CTL) for 2 weeks. (**A**) Body weight monitoring; (**B**) Adipose tissue weight; (**C**) Representative images of Hematoxylin/Eosin staining of paraffin sections of adipose tissues. Size bar = 40 μm; (**D**) Adipocyte size analysis; (**E**) Intraperitoneal glucose tolerance test (GTT). (*n* = 6 per condition, means ± S.E.M, *t*-test, * *p* < 0.05, ** *p* < 0.01, *** *p* < 0.001).

An HFD-induced obesity mouse model was applied to validate the anti-obesity effect of EGCG. After feeding of HFD for 8 weeks, EGCG was administrated to C57BL/6 mice under continuous HFD feeding for 2 weeks. EGCG was detected in the mouse plasma samples (Figure S1). As indicated in Figure 1, EGCG treatment reduced body weight by 10% and gonadal WAT (gWAT) mass by 20% (Figure 1A,B). H&E staining indicated that EGCG reduced adipocyte size in inguinal WAT (iWAT) and gWAT (Figure 1C,D). In the intraperitoneal glucose tolerance test, glucose tolerance was ameliorated in the EGCG treated group compared to the vehicle group (Figure 1E).

### 3.2. EGCG Increased Autophagy in Gonadal WAT

**Figure 2:**
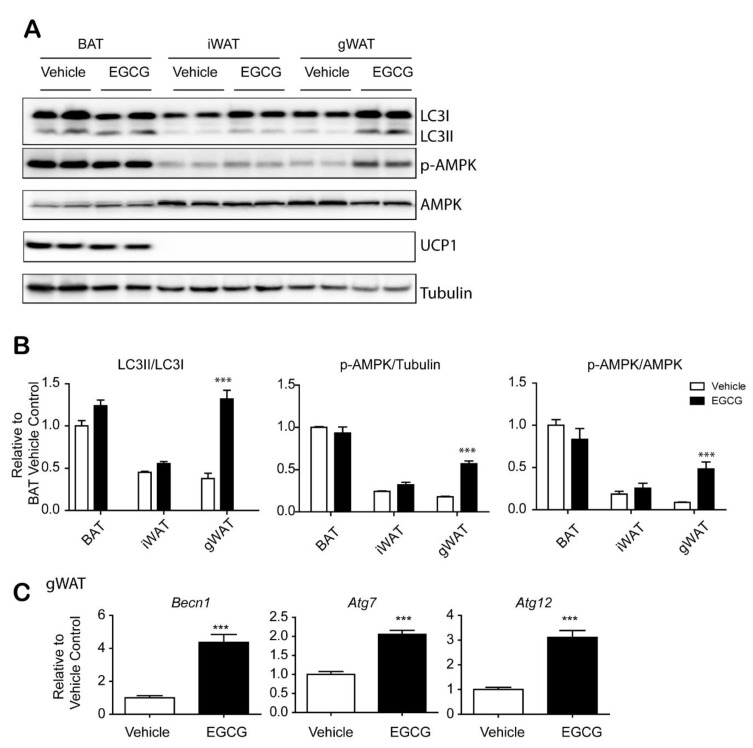
Epigallocatechin-3-gallate (EGCG) treatment increases autophagy in visceral white adipose tissue. (**A**) Immunoblot analysis of brown adipose tissue (BAT), inguinal white adipose tissue (iWAT) and gonadal white adipose tissue (gWAT) from mice fed a high-fat diet (HFD) for 8 weeks and then treated with EGCG or vehicle for 2 weeks; (**B**) Quantification of (A); (**C**) qPCR analysis of genes related to autophagy in gWAT. (*n* = 6 per condition, means ± S.E.M., *t*-test, *** *p* < 0.001).

We previously reported that EGCG increases autophagy in adipocytes cultured in vitro. Immunoblot analysis of the LC3II/LC3I ratio showed the relationship between EGCG and autophagic flux in adipose tissue (Figure 2A,B). The results indicated that EGCG treatment increased autophagic flux in WAT, but not in brown adipose tissue (BAT). The elevated LC3II/LC3I ratio was associated with an increase in the p-AMPK/AMPK and p-ULK1/ULK1 ratios in WAT. There was no effect on the expression level of brown adipocyte marker, uncoupling protein 1 (UCP1), by EGCG treatment. Moreover, we examined the expression levels of genes related to autophagy by qPCR and found that *Becn1, Atg7* and *Atg12* in gWAT were increased by EGCG administration (Figure 2C). These data suggested that the treatment of EGCG increased autophagy in a WAT-specific manner, potentially by the AMPK-mediated signaling pathway.

### 3.3. EGCG Regulated the AMPK Downstream Targets, ATGL and SREBP1-c, in WATs

**Figure 3:**
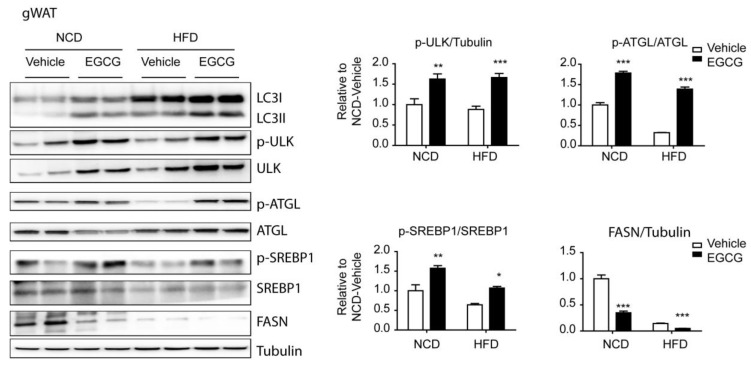
Effects of Epigallocatechin-3-gallate (EGCG) on phosphorylation levels of AMP-activated protein kinase (AMPK) downstream proteins in gonadal white adipose tissue (gWAT). Immunoblot analysis of gWAT from mice fed a normal chow diet (NCD) or high-fat diet (HFD) for 8 weeks and then treated with EGCG or vehicle for 2 weeks. (*n* = 6 per condition, means ± S.E.M., *t*-test, * *p* < 0.05, ** *p* < 0.01, *** *p* < 0.001).

**Figure 4:**
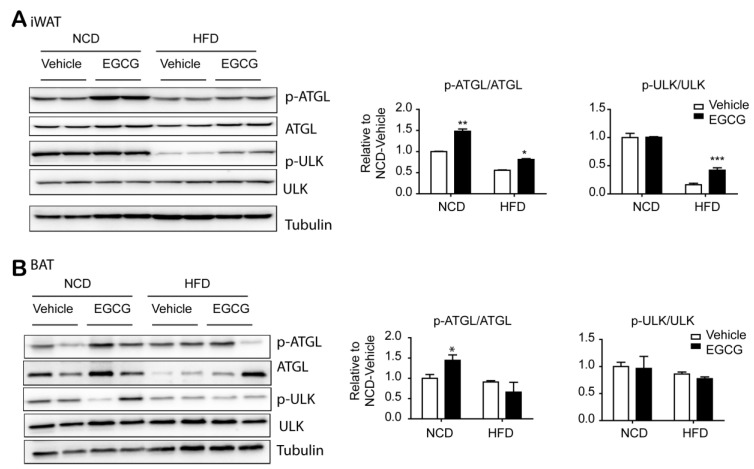
Effects of Epigallocatechin-3-gallate (EGCG) on phosphorylation levels of AMP-activated protein kinase (AMPK) downstream proteins in inguinal white adipose tissue (iWAT) and brown adipose tissue (BAT). (**A**,**B**) Immunoblot analysis of (**A**) iWAT and (**B**) BAT from mice fed a normal chow diet (NCD) or high-fat diet (HFD) for 8 weeks and then treated with EGCG or vehicle for 2 weeks. (*n* = 6 per condition, means ± S.E.M., *t*-test, * *p* < 0.05, ** *p* < 0.01, *** *p* < 0.001).

Cytosolic lipolysis is a well-known pathway of triglyceride catabolism. AMPK upregulates lipolysis by phosphorylating ATGL at Ser406. Immunoblot analysis indicated that EGCG treatment increased expression and phosphorylation levels of ATGL in iWAT and gWAT, but not in BAT (Figure 3 and Figure 4A,B). This WAT-specific upregulation was prominent in gWAT. In addition, EGCG treatment increased the phosphorylation of sterol regulatory element-binding protein 1c (SREBP1-c) at Ser372 and reduced fatty acid synthase (FASN) levels. These data suggested that treatment of EGCG had WAT-specific effects in increasing lipid catabolism, potentially by AMPK-dependent regulation of lipolysis and lipogenesis.

### 3.4. The Anti-Obesity Effects of EGCG Required Beclin1 Expression in Adipose Tissue

**Figure 5:**
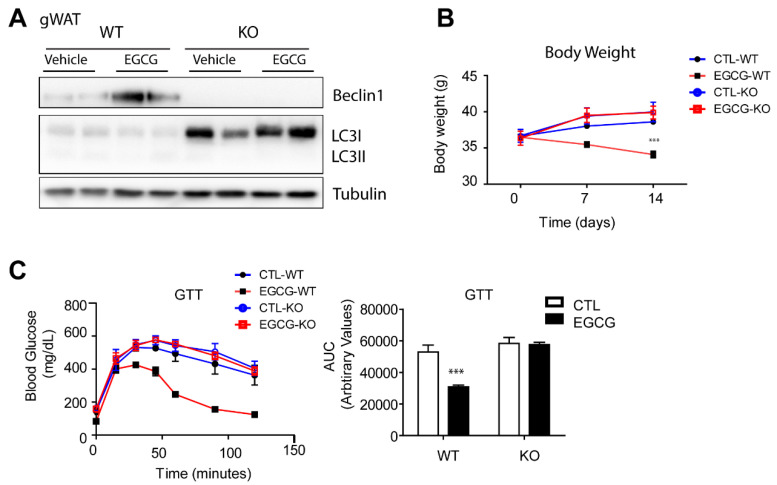
Adipocyte-specific Beclin1 knockout (KO) attenuates the effects of Epigallocatechin-3-gallate (EGCG) on body weight and glucose tolerance. For 8 weeks, wild type (WT) and adipocyte-specific Beclin1 KO mice were fed a high-fat diet (HFD) and then treated with EGCG (20 mg/kg/day) (EGCG-WT, EGCG-KO) or vehicle (CTL-WT, CTL-KO) for 2 weeks. (**A**) Immunoblot analysis of gonadal white adipose tissue (gWAT) of WT and KO mice; (**B**) Body weight monitoring; (**C**) Intraperitoneal glucose tolerance test (GTT). (*n* = 6 per condition, means ± S.E.M., *t*-test, *** *p* < 0.001).

To confirm whether the effects of EGCG contribute to activating autophagy, we utilized an adipocyte-specific Beclin1 KO mouse model. Beclin1 is a core protein necessary for autophagosome formation; it is required for initiating the recruitment of autophagic machinery. Adipocyte-specific Beclin1 KO was confirmed in gWAT by Immunoblot analysis (Figure 5A). Consistent with previous reports, adipocyte-specific Beclin1 KO mice demonstrated the accumulation of LC3 due to defects in the autophagic process. Adipocyte-specific Beclin1 KO mitigated the effects of EGCG on visceral WAT mass, glucose tolerance and energy expenditure, indicating that the anti-obesity effect of EGCG required Beclin1-dependent autophagy (Figure 5B,C). Collectively, our data illustrated that EGCG activates the anti-obesity effect under the regulation of autophagy-related lipid catabolism in WATs.

## 4. Discussion

EGCG is known to possess various health benefits, including the prevention of cancer [20], cardiovascular diseases [5], and inflammation [14]. In addition, EGCG has been proposed as useful in the prevention and treatment of obesity-associated metabolic disorders [5]. However, the molecular mechanisms of the anti-obesity effects of EGCG and its specific roles in adipose lipid metabolism remain unclear.

Based on our previous in vitro studies [15], we hypothesized that EGCG upregulates autophagic flux in adipose tissue. Our data indicated that EGCG increased autophagy in WAT, AMPK signaling and phosphorylation of ULK1. ULK1 is an autophagy-initiating kinase that phosphorylates Beclin1 to induce autophagy [13]. We used adipocyte-specific Beclin1 KO mice to test whether Beclin1-dependent autophagy takes part in the effect of EGCG. The results demonstrated that the anti-obesity effect of EGCG was absent in Beclin1 KO mice, suggesting that Beclin1 is one of the molecular players causing the EGCG effect. Although Beclin1 is a core molecule that initiates autophagy [21], it is also involved in regulating apoptosis and cell death [21]. Further studies will be required to demonstrate whether EGCG targets Beclin1 regardless of its roles in autophagy.

In our study, we revealed that EGCG regulated lipid catabolism through AMPK-mediated mechanisms, which is one of the anti-obesity mechanisms of EGCG in adipocytes. For example, EGCG increased phosphorylation levels of the downstream targets of AMPK, ATGL and SREBP1-c, increasing lipolysis and suppressing lipogenesis. In this regard, the previous studies have reported that EGCG-mediated AMPK activation involves an enhanced cellular production of reactive oxygen species (ROS) by the pro-oxidant action of EGCG in various cell types including adipocytes [6], hepatocytes [22] and cancer cells [23]. Thus, we speculated that the in vivo anti-obesity effect of EGCG mediated ROS-dependent AMPK activation that suppresses genes involved in adipogenesis [6] and lipogenesis [24], and stimulates those involved in lipolysis [25]. Although the regulatory mechanisms of lipolysis [11] and lipogenesis [12] by AMPK are well accepted, further loss/gain of function studies are required for further explanation of the molecular players in EGCG-induced AMPK activation and lipid catabolism.

A critical finding of our study is that EGCG induced lipid catabolism in a visceral WAT-specific manner. It is well accepted that the accumulation of visceral WAT belongs to the pathogenesis of insulin resistance and diabetes, while subcutaneous WAT is beneficial for insulin sensitivity and to maintain energy homeostasis. In this regard, the waist-to-hip ratio has been used to determine abdominal obesity as a potential indicator of obesity-related serious health conditions. Although we did not investigate molecular mechanisms of the visceral adipose tissue-specific effect of EGCG, knowledge of this mechanism will help in developing strategies to target visceral fat for metabolic health.

Modifying effects of EGCG on gut microbiota has been proposed as one of the potential mechanisms of anti-obesity activity of EGCG [26]. Moreover, recent studies have demonstrated that several microbial metabolites of EGCG generated in the gastrointestinal tract are absorbed into the systemic circulation [27,28] and effectively improve insulin sensitivity and glucose tolerance in rodent models [29]. Although we did not examine the levels of the metabolites in plasma or tissues, we speculated that the microbial metabolites of EGCG might contribute to the anti-obesity effects of EGCG treatment. Further studies will be necessary to understand the pharmacokinetics and pharmacodynamics of EGCG and its metabolites in relation to anti-obesity effects of EGCG. Particularly, it will be necessary to establish the dose-response relationship of EGCG and its metabolites in the physiologically relevant concentration range.

In addition, a reduction in postprandial glucose levels by EGCG is associated with inhibition of alpha-amylase [30]. Although it was not investigated in the current study, we do not exclude the possibility that non-absorbed EGCG affects gut microbiota and digestive enzymes in gastrointestinal tracts. Further investigation into the distinct contribution of non-absorbed and absorbed EGCG will provide valuable information on molecular mechanisms of action of EGCG for the improvement of metabolic profiles.

In summary, our results showed that EGCG reduced visceral adiposity in a diet-induced obesity mouse model by activating autophagy and lipolysis in WAT through an AMPK-mediated mechanism. Furthermore, the adipocyte-specific Beclin1 KO mouse model demonstrated that the induction of Beclin1-dependent autophagy was required for the anti-obesity effects of EGCG. In this sense, EGCG is a potential candidate for preventing or treating obesity; however, further investigations will be necessary to determinethe molecular mechanisms of EGCG in targeting visceral WAT.

## Supplementary Materials

The following are available online at https://www.mdpi.com/2072-6643/12/10/3072/s1, Table S1: qPCR Primers for mRNAs, Supplemental Methods: Measurement of EGCG, Figure S1: Measurement of EGCG in plasma obtained from mice treated with EGCG.
