# Supplementary material for: Epigallocatechin-3-Gallate Reduces Visceral Adiposity Partly through the Regulation of Beclin1-Dependent Autophagy in White Adipose Tissues
Source: Nutrients. 2020 Oct 8;12(10):3072. doi: 10.3390/nu12103072 (PMC7600517; doi:10.3390/nu12103072)
Supplement: Supplementary file 1 [file nutrients-12-03072-s001.pdf]

Table S1. qPCR Primers for mRNAs

Genes	Forward (5′→3′)	Reverse (5′ →3′)
Beclin1	GCTGGAGTTGGATGACGAAC	GCAAGCGACCCAGTCTGAA
ATG 7	CTGACCTTCGCGGACCTAAA	GGTCCCCGGATTAGAGGGAT
ATG 12	CCCCAGACCAAGAAGTTGGAA	CCATGCCTGGGATTTGCAGTA

Supplemental Methods

Measurement of EGCG

Control plasma samples without EGCG were spiked with a serial amount of EGCG standard solution. All plasma samples were introduced with 10 μL of preservative solution (20% L-ascorbic acid and 0.05% Na2EDTA) and extracted with 1000 μL of ethyl acetate containing with 5 ng mL-1 of EGC as an internal standard. Supernatant was collected after vortexing and centrifugation (13000 rcf, 4°C, 10min) and subsequently evaporated using nitrogen purge. The residues were reconstituted in 50 μL of methanol. Next, 5 μL of sample solution was injected into 1290 UPLC system (Agilent, CA, USA) and analyzed by Triple Quadrupole Mass Spectrometer (Agilent 6460 QqQ MS, Agilent, CA, USA).

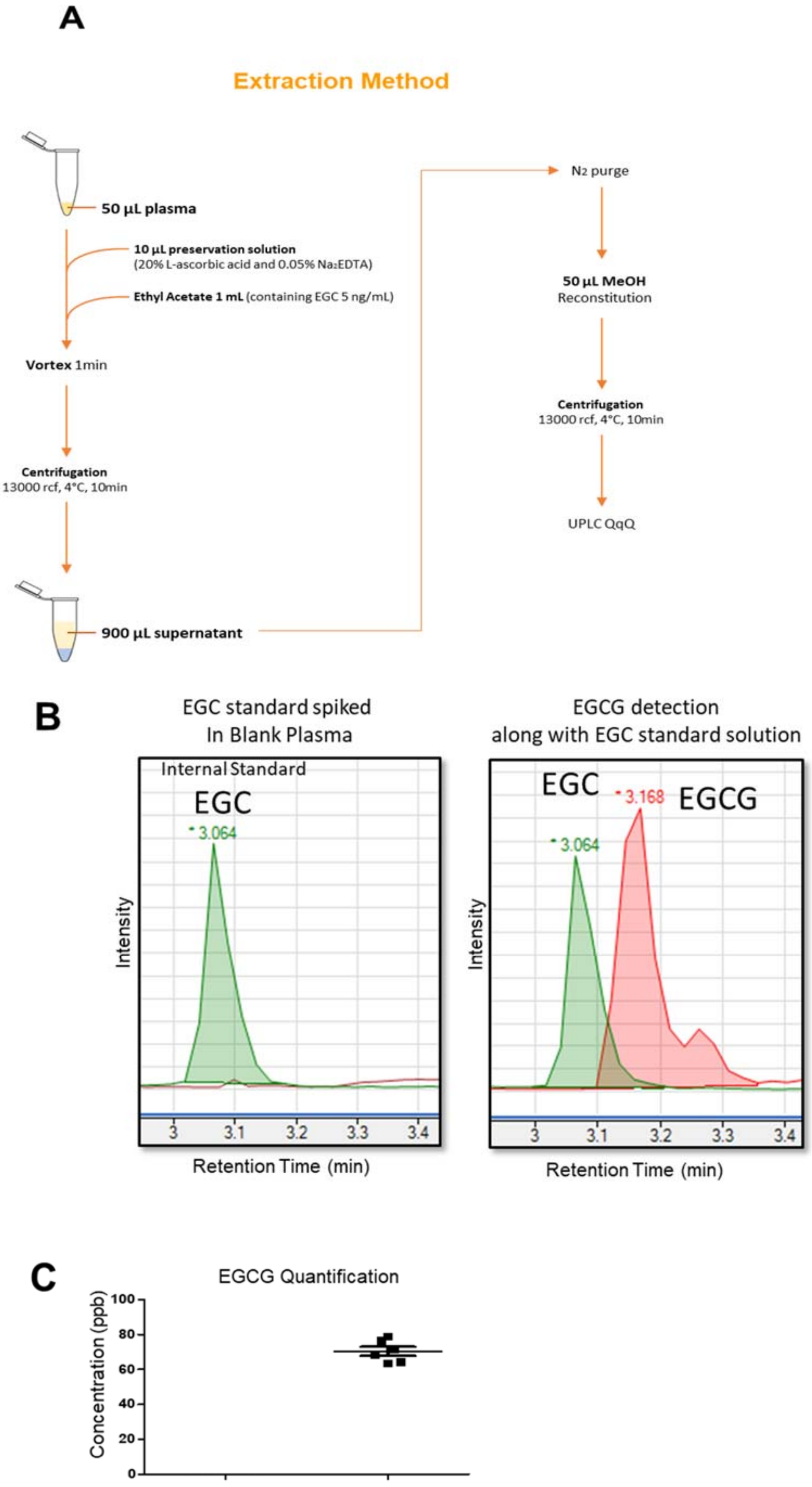

Figure S1. Measurement of EGCG in plasma obtained from mice treated with EGCG

A. Schematic diagram of an extraction process from plasma samples for EGCG detection

B. Representative chromatogram of EGCG and EGC. EGC was used as an internal standard.

C. Concentration of EGCG in plasma samples obtained from mice treated with EGCG (20mg/kg/day, p.o.) for 14 days and vehicle controls.
